# Does the perception of neighborhood built environmental attributes influence active transport in adolescents?

**DOI:** 10.1186/1479-5868-10-38

**Published:** 2013-03-26

**Authors:** Femke De Meester, Delfien Van Dyck, Ilse De Bourdeaudhuij, Benedicte Deforche, Greet Cardon

**Affiliations:** 1 Department of Movement and Sport Sciences, Faculty of Medicine and Health Sciences, Ghent University, Watersportlaan 2, Ghent, B-9000, Belgium; 2 Department of Human Biometry and Biomechanics, Faculty of Physical Education and Physiotherapy, Vrije Universiteit Brussel, Brussels, Belgium; 3 Research Foundation Flanders (FWO), Brussels, Belgium

**Keywords:** Active transport, Adolescents, Perceived neighborhood environmental factors

## Abstract

**Background:**

Among Belgian adolescents active transport (AT) is a common physical activity (PA) behavior. Preliminary evidence suggests that AT can be an important opportunity for increasing adolescents’ daily PA levels. To inform interventions, predictors of this PA behavior need to be further explored. Therefore, in the perspective of the ecological models this study aimed (a) to investigate the relationship between the perception of neighborhood built environmental attributes and adolescents’ AT and (b) to explore the contribution of the perception of neighborhood built environmental attributes beyond psychosocial factors.

**Methods:**

For the purpose of this study, data from the Belgian Environmental Physical Activity Study in Youth (BEPAS-Y), performed between 2008 and 2009, was used. The final study population consisted of 637 adolescents aged 13–15 years. The participants completed a survey measuring demographic and psychosocial factors, the Flemish Physical Activity Questionnaire and the Dutch version of the Neighborhood Environmental Walkability Scale.

**Results:**

A set of stepwise linear regression analyses with backward elimination revealed that a shorter distance to school, perceiving neighborhoods to have connected streets, a lower degree of land use mix diversity, less infrastructure for walking and a lower quality of the infrastructure for walking are associated with more min/day AT to and from school (p all <0.05). Furthermore, marginally significant associations (p < 0.10) were found between residential density and safety from crime and AT to and from school. No relationship between the perception of the neighborhood built environmental attributes and walking for transport during leisure time and cycling for transport during leisure time was found.

**Conclusions:**

The substantial contribution of the perception of neighbourhood built environmental attributes to AT found in Belgian adults, could not totally be confirmed by this study for Belgian adolescents. Among Belgian adolescents, the contribution of neighborhood environmental perceptions to explain the variance in AT seems to be dependent of the purpose of AT. Further research is needed to explore this relationship in specific subgroups and to overcome some of the limitations this study had to contend with.

## Introduction

Walking and cycling for transport, also called “active transport” (AT), are common physical activity (PA) behaviors among Belgian adolescents. AT has been acknowledged by a number of studies as a PA behavior with a significant contribution to the overall PA level [1,2]. Given the health benefits related to PA [3-5], the proportion of school-aged youth that does not achieve the public health recommendations for PA [6,7] and the decline in time spent in PA during adolescence [8,9] it is of utmost importance to understand the factors that influence adolescents’ PA behavior and in particular adolescents’ AT to develop effective interventions.

Ecological models postulate the importance of the environment to explain PA behavior [10]. In adults, neighborhood built environmental attributes (e.g. neighborhood walkability, typically characterized by land use mix, connectivity, residential density) have been found to be related to PA, and in particular to AT, in different continents and countries [11-14].

In contrast to the consistent results in adults, the association between neighborhood built environmental attributes and adolescents’ levels of PA is rather blurred [15,16]. Within this area, more attention has been given to environmental correlates of AT to school than to other forms of AT [17,18]. A number of studies among youth have found that higher levels of AT are associated with a shorter distance to school, higher residential density, the presence of facilities, safe roads, mixed land use, the presence of cul-de sacs and the presence of facilities to assist active travel [17,19]. Nevertheless, based on the existing evidence, no definite conclusions can be drawn concerning the contribution of neighborhood built environmental factors in explaining other types of AT in adolescents, like AT during leisure time or to other destinations besides school [17,20].

The inconsistencies between results of previous studies are potentially influenced by the different definitions of the neighborhood built environmental attributes [21] and the different methods that were used to measure PA and the built environmental attributes [15,16,22]. According to Ball et al. (2008), it is possible that objective assessments of built environmental attributes are indirectly associated with PA, whereas perceptions of environmental attributes have a more direct influence on PA [23]. Thus, the presence of neighborhood environmental attributes might not automatically influence the behavior in the absence of awareness of those attributes [23].

Furthermore, given the large variation in built environmental attributes between different parts of the world (e.g. US, Australia and Europe), the conclusions from studies conducted in the US and Australia may not be generalizable to European countries. Finally, an important premise of the ecological models is that the contribution of demographic, psychosocial or environmental factors in explaining PA cannot be seen on their own [10]. When investigating the relative influence of environmental factors on PA, demographic and psychosocial factors need to be taken into account.

Regarding the inconsistencies and gaps in this area the present study investigated the relationship between the perception of neighborhood built environmental attributes and adolescents’ AT. Secondly, this study explored the contribution of the perception of neighborhood built environmental attributes beyond psychosocial factors.

## Method

### Procedure and sample

The Belgian Environmental Physical Activity Study in Youth (BEPAS-Y) was conducted in Ghent (Belgium). The design, methods of recruitment, sampling and data collection are described elsewhere [24]. In short, 637 adolescents were recruited (response rate 59,0%; 49.5% boys) from 32 neighborhoods differing in objectively determined neighborhood walkability (GIS-based) and socio-economic status (SES) (using Belgian census data derived from the Belgian National Institute of Statistics): 16 low SES neighborhoods and 16 high SES neighborhoods of which in each SES group 8 were high walkable and 8 were low walkable. Adolescents who consented to participate were asked to fill in a questionnaire regarding PA, perceived environmental attributes, demographic and psychosocial factors.

### Measures

The Flemish Physical Activity Questionnaire (FPAQ; interview version) was used [25] to determine the duration (hours and minutes per day) of AT to and from school, walking for transport during leisure time and cycling for transport during leisure time. The computerized version of this questionnaire was found to be a reliable and a reasonable valid instrument for the assessment of different dimensions of physical activity in 12 to 18 year old adolescents [25]. The FPAQ has been used in previous research assessing PA among adolescents [26,27].

To measure perceived neighborhood built environmental attributes, the Dutch version of the Neighborhood Environmental Walkability Scale (NEWS) was used [28]. This questionnaire was designed to assess the perception of neighborhood environmental attributes that were found to be relevant to walking and cycling for transport, based on the transportation and urban planning literature [29].

To assess demographic factors (adolescents’ gender, age and SES: educational attainment and employment of the parents) and psychosocial factors (modeling, social norm, social support from family and friends, self-efficacy, perceived benefits and perceived barriers towards PA) questions derived from previous studies in adults and adolescents [30-34] were used.

### Analyses

Descriptive statistics were computed for the AT variables, demographic characteristics, psychosocial factors and explanatory variables (perceived neighborhood built environmental attributes) using SPSS 17.0. Tests for normal distribution revealed positive skewness of the AT variables. To obtain distributions that more closely approximated symmetry, logarithmic transformations were conducted and the transformed variables were used in the analyses. For ease of interpretation, summary data of untransformed AT variables are reported in minutes/day (Table 1).

**Table 1:** Descriptive statistics of the demographic characteristics, psychosocial variables and perceived built environmental attributes

	**Content of the item**	**Response category**	**Descriptives**
***Demographic characteristics***			
**Age: mean (SD)**			14.5 (0.9)
**Gender: %**			
Male			49.5
Female			50.5
**Employment status: %**			
Both employed			68.5
One parent unemployed			26.7
Both unemployed			4.9
**Educational level: %**			
**Mother:**			
Less than high school			9.9
Completed high school			25.1
Completed college			40.4
Completed University			24.6
**Father:**			
Less than high school			7.5
Completed high school			36.0
Completed college			26.6
Completed University			29.8
***Psychosocial variables: mean (SD)***			
Modeling (2 items)	How frequently participate family and friends in PA?	5-point scale^a^	3.6 (0.8)
Social norm (2 items)	Does the family and friends think that they should participate regularly in PA?	5-point scale^b^	3.5 (1.1)
Social support from family (3 items)	Social support from family towards PA (e.do PA together, invite to do PA together, encourage to do PA)	5-point scale^c^	2.6 (0.9)
Social support from friends (3 items)	Social support from friends towards PA (e.do PA together, invite to do PA together, encourage to do PA)	5-point scale^c^	3.0 (0.9)
Self-efficacy towards internal barriers (7 items)	Confidence to do PA under potentially difficult situations (internal: e.g. feeling stressed, being unwell	5-point scale^d^	3.5 (0.8)
Self-efficacy towards external barriers (6 items)	Confidence to do PA under potentially difficult situations (external: e.g. after a long and exhausting day, early in the morning)	5-point scale^d^	3.3 (0.9)
Perceived benefits towards PA (18 items)	Agreement with possible positive effects of PA (e.g. losing weight, having fun)	5-point scale^b^	3.7 (0.6)
Perceived barriers towards PA (26 items)	Agreement with possible barriers, preventing the adolescent to do PA (e.g. external obstacles, lack of time, lack of interest)	5-point scale^c^	2.1 (0.6)
***Perceived neighborhood environmental attributes: mean (SD)***			
Residential density (5 items)	Presence of different types of residences (e.g. detached single family residences, row houses, apartments	5-point scale ^e^	155.5 (45.2)
Land use mix diversity (22 items)	Distance to local facilities (e.g. supermarket, post office, park, library)	5-point scale ^f^	3.0 (0.8)
Land use mix access (4 items)	Access to neighborhood services (e.g. ease to walk to public transport, possibilities to do shopping in local area)	4-point scale ^g^	3.4 (0.6)
Distance to school (1item)	Distance to the school of the adolescent	5-point scale ^f^	2.30 (1.37)
Connectivity (3 items)	Connectedness of street network (e.g. presence of intersections, dead-end streets, alternate routes)	4-point scale^g^	2.9 (0.6)
Walking infrastructure (4 items)	Availability and quality of walking infrastructure (e.g. footpaths on most streets, maintenance of footpaths, footpaths separated from streets)	4-point scale^g^	2.9 (0.8)
Cycling infrastructure (5 items)	Availability and quality of cycling infrastructure (e.g. cycling lanes in most streets, maintenance of cycling lanes, cycling lanes separated from streets)	4-point scale^g^	2.2 (0.7)
Safety for cycling (2 items)	Prevalence of bicycle theft and precautionary measures against bicycle theft	4-point scale^g^	2.6 (0.8)
Aesthetics (4 items)	Presence of aesthetic features (e.g. green spaces, attractive buildings, streets free from litter and graffiti)	4-point scale^g^	2.6 (0.6)
Safety for traffic (8 items)	Perceived safety from traffic problems (speed of traffic in neighborhood, availability of pedestrian crossings and traffic signals, exhaust fumes from cars)	4-point scale^g^	2.8 (0.5)
Safety for crime (5 items)	Perceived safety from crime (e.g. crime prevalence in the neighborhood, perceived safety from walking and cycling during the day and night)	4-point scale^g^	3.4 (0.6)
Convenience of recreation facilities (18 items)	Distance to PA facilities (e.g. soccer field, squash court, running track, swimming pool)	5-point scale^f^	2.9 (0.8)
***Active transport variables:***			
Active transport to and from school: mean min/day (SD)			11.5 (14.5)
Active transport to and from school: % that uses active transport to go to school			54.5
Walking for transport during leisure time: mean min/day (SD)			9.7 (11.7)
Walking for transport during leisure time: % that walks for transport during leisure time			65.8
Cycling for transport during leisure time: mean min/day (SD)			8.2 (10.7)
Cycling for transport during leisure time: % that cycles for transport during leisure time			63.5

PA: physical activity.

^a^ never or a few times a year, monthly, more than once a month, more than once a week, almost daily.

^b^ strongly disagree, somewhat disagree, neither agree or disagree, somewhat agree, strongly agree.

^c^ never, seldom, sometimes, often, very often^d^ I know I can’t do it, I think I can’t do it, I don’ know If I can do it, I think I can do it, I know I can do it.

^e^ none, a few, about half, a lot, all.

^f^ > 30 min, 21–30 min, 11–20 min, 6–10 min, 1–5 min,

^g^ Strongly disagree, somewhat disagree, somewhat agree, strongly agreeNote: all perceived built environmental attributes were positively scored: higher score = more walkable.

Stepwise linear regression analyses with backward elimination were used to examine associations between the dependent variables (min/day AT to and from school, walking for transport during leisure time and cycling for transport during leisure time) and the explanatory variables (e.g. the perceived neighborhood built environmental attributes).

First, multicollinearity among demographic characteristics, psychosocial factors and neighborhood built environmental attributes was checked by conducting Pearson’s correlations in SPSS 17.0. When the magnitude of the correlation coefficient indicated multicollinearity (>0.60), the variable that correlated the least with the dependent variable was excluded from further analyses.

All models were controlled for a set of demographic factors (age, gender, parental employment and educational attainment of the mother or father), regardless of their relationship with the AT-variables. These demographic covariates were entered as the first block in all models (model 1). To investigate the association between AT and the perception of neighborhood built environmental attributes, the neighborhood built environmental attributes were entered as the second block in the models. All neighborhood environmental attributes were entered simultaneously and the attributes that met the criterion for elimination (p ≥ 0.10) were sequentially removed. The neighborhood environmental attribute that was associated the least with the dependent variable was considered the first for removal. This procedure was repeated until the neighborhood environmental attributes with a p < 0.10 remained (model 2).To explore the contribution of the perception of neighborhood built environmental attributes beyond psychosocial factors, the psychosocial variables (modeling, social norm, social support from family and friends, self-efficacy, perceived benefits and perceived barriers towards PA) were entered as the second block, regardless of their relationship with the AT-variables. Again, all neighborhood built environmental attributes were entered simultaneously and the attributes that met the criterion for elimination (p ≥ 0.10) were sequentially removed until the neighborhood environmental attributes that did not meet the criterion for removal remained (model 3). Clustering of individuals in neighborhoods was taken into account by using multilevel modeling (two levels: adolescent – neighborhood).

## Results

Descriptive data for the demographic characteristics, psychosocial factors, perception of neighborhood built environmental attributes and AT variables can be found in Table 1.

### Environmental correlates of active transport to and from school

Table 2 shows the results of the stepwise linear regression analyses with backward elimination concerning AT to and from school. The regression analysis with the demographic covariates entered as a first block, followed by the neighborhood built environmental attributes (model 2), revealed that distance to school (p < 0.001) and connectivity (p = 0.011) showed a strong and significant positive association with AT to and from school, whereas land use mix diversity (p = 0.042) and walking infrastructure (p = 0.032) showed a negative association with AT to and from school. Both residential density (p = 0.065) and safety for crime (p = 0.070) were found to be positively but marginally significantly associated with AT to and from school. Land use mix access, cycling infrastructure, safety for cycling, aesthetics and safety for traffic were not significant and excluded from the final model.

**Table 2:** Results of the stepwise linear regression analyses with backward elimination concerning active transport to and from school

	**Active transport to and from school**								
	**MODEL 1**			**MODEL 2**			**MODEL 3**		
***Demographic characteristics***	ß (SE)	95% CI	p	ß (SE)	95% CI	p	ß (SE)	95% CI	p
Age (yrs)	0.032(0.030)	−0.027-0.091	0.277	0.062(0.031)	0.001-0.123	0.045	0.061(0.031)	0.000-0.122	0.050
Gender (ref: male)	0.104(0.053)	0.000-0.208	0.049	0.068(0.054)	−0.038-0.174	0.206	0.067(0.056)	−0.043-0.177	0.228
Parental employment									
*Both unemployed (ref)*									
*One parent unemployed*	0.263(0.145)	−0.021-0.547	0.070	0.321(0.155)	0.017-0.625	0.039	0.283(0.155)	−0.021-0.587	0.067
*Both employed*	0.403(0.143)	0.123-0.683	0.005	0.392(0.152)	0.094-0.690	0.010	0.377(0.150)	0.083-0.671	0.012
Educational attainment mother									
*Less than high school (ref)*									
*Completed high school*	−0.128(0.106)	−0.336-0.080	0.229	−0.147(0.110)	−0.363-0.069	0.182	−0.119(0.111)	−0.337-0.099	0.284
*Completed college*	0.001(0.105)	−0.205-0.207	0.999	−0.011(0.109)	−0.225-0.203	0.916	0.059(0.109)	−0.155-0.273	0.590
*Completed University*	0.004(0.111)	−0.214-0.222	0.975	−0.057(0.115)	−0.282-0.168	0.619	−0.059(0.117)	−0.288-0.170	0.616
Educational attainment father									
*Less than high school (ref)*									
*Completed high school*									
*Completed college*									
*Completed University*									
***Psychosocial factors***	ß (SE)	95% CI	p	ß (SE)	95% CI	p	ß (SE)	95% CI	p
Modeling							0.046(0.038)	−0.028-0.120	0.226
Social norm							−0.026(0.028)	−0.081-0.029	0.350
Social support from family							0.058(0.035)	−0.011-0.127	0.099
Social support from friends							0.026(0.033)	−0.039-0.091	0.428
Self-efficacy internal							0.084(0.045)	−0.004-0.172	0.059
Self-efficacy external									
Perceived benefits							−0.112(0.052)	−0.224-(−0.020)	0.030
Perceived barriers							0.035(0.058)	−0.079-0.149	0.550
***Perceived neighborhood environmental attributes***	ß (SE)	95% CI	p	ß (SE)	95% CI	p	ß (SE)	95% CI	p
Residential density				0.001(0.001)	−0.001-0.003	0.065	0.001(0.001)	−0.001-0.003	0.089
Land use mix diversity				−0.099(0.048)	−0.193-(−0.005)	0.042	−0.112(0.049)	−0.208-(−0.016)	0.021
Land use mix access									
Distance to school				0.165(0.024)	0.118-0.212	<0.001	0.164(0.024)	0.117-0.211	<0.001
Connectivity				0.142(0.056)	0.032-0.252	0.011	0.116(0.057)	0.004-0.228	0.043
Walking infrastructure				−0.096(0.044)	−0.182-(−0.010)	0.032	−0.091(0.045)	−0.179-(−0.003)	0.042
Cycling infrastructure									
Safety for cycling									
Aesthetics									
Safety for traffic									
Safety for crime				0.092(0.051)	−0.008-0.192	0.070	0.097(0.052)	−0.005-0.199	0.062

Note: Due to multicollinearity between the variables “educational attainment mother” and “educational attainment father”, “self-efficacy internal” and “self-efficacy external” and “land use mix diversity” and “land use mix access”, the variables “educational attainment father”, “self-efficacy external” and “land use mix access” were excluded from further analyses. CI indicates confidence interval.

The results of the regression analyses with the psychosocial covariates entered as a second block (model 3) were similar to the results of model 2. Distance to school (p < 0.001) and connectivity (p = 0.043) showed a significant positive association with AT to and from school, whereas land use mix diversity (p = 0.021) and walking infrastructure (p = 0.042) showed a negative association with AT to and from school. Residential density (p = 0.089) and safety for crime (p = 0.062) were found to be positively but marginally associated with AT to and from school. Land use mix access, cycling infrastructure, safety for cycling, aesthetics and safety for traffic were not significant and excluded from the final model.

### Environmental correlates of walking for transport during leisure time

Table 3 shows the results of the stepwise linear regression analyses with backward elimination concerning walking for transport during leisure time. The regression analysis with the demographic covariates entered as a first block, followed by the neighborhood built environmental attributes (model 2), revealed that higher levels of traffic safety were significantly associated (p = 0.040) with more min/day walking for transport during leisure time. Traffic safety remained significant (p = 0.023) after adding the set of psychosocial covariates (model 3). Residential density, land use mix diversity, land use mix access, connectivity, walking infrastructure, cycling infrastructure, safety for cycling, aesthetics, safety for crime and convenience of recreation facilities were not significant and excluded from the final models.

**Table 3:** Results of the stepwise linear regression analyses with backward elimination concerning walking for transport during leisure time

	**Walking for transport during leisure time**								
	**MODEL 1**			**MODEL 2**			**MODEL 3**		
***Demographic characteristics***	ß (SE)	95% CI	p	ß (SE)	95% CI	p	ß (SE)	95% CI	p
Age (yrs)	0.013(0.025)	−0.036-0.062	0.600	0.009(0.025)	−0.040-0.058	0.724	0.002(0.025)	−0.047-0.051	0.950
Gender (ref: male)	−0.087(0.044)	−0.173-(−0.001)	0.046	−0.091(0.044)	−0.177-(−0.005)	0.039	−0.081(0.046)	−0.171-0.009	0.078
Parental employment									
*Both unemployed (ref)*									
*One parent unemployed*	−0.151(0.123)	−0.392-0.090	0.218	−0.159(0.123)	−0.400-0.082	0.197	−0.154(0.127)	−0.403-0.095	0.225
*Both employed*	−0.196(0.121)	−0.433-0.041	0.107	−0.198(0.121)	−0.435-0.039	0.106	−0.178(0.125)	−0.423-0.067	0.154
Educational attainment mother									
*Less than high school (ref)*									
*Completed high school*	−0.229(0.088)	−0.401-(−0.057)	0.009	−0.229(0.089)	−0.403-(−0.055)	0.010	−0.239(0.091)	−0.417-(−0.061)	0.008
*Completed college*	−0.259(0.087)	−0.430-(−0.088)	0.003	−0.263(0.088)	−0.435-(−0.091)	0.003	−0.265(0.089)	−0.439-(−0.091)	0.003
*Completed University*	−0.315(0.093)	−0.497-(−0.133)	<0.001	−0.328(0.094)	−0.512-(−0.144)	<0.001	−0.324(0.096)	−0.512-(−0.136)	<0.001
Educational attainment father									
*Less than high school (ref)*									
*Completed high school*									
*Completed college*									
*Completed University*									
***Psychosocial factors***	ß (SE)	95% CI	p	ß (SE)	95% CI	p	ß (SE)	95% CI	p
Modeling							0.019(0.031)	−0.042-0.080	0.538
Social norm							−0.006(0.023)	−0.051-0.039	0.780
Social support from family							−0.017(0.028)	−0.072-0.038	0.559
Social support from friends							0.056(0.027)	0.003-0.109	0.038
Self-efficacy internal									
Self-efficacy external							−0.074(0.030)	−0.133-(−0.015)	0.014
Perceived benefits							0.025(0.041)	−0.055-0.105	0.510
Perceived barriers							−0.003(0.044)	−0.089-0.083	0.950
***Perceived neighborhood environmental attributes***	ß (SE)	95% CI	p	ß (SE)	95% CI	p	ß (SE)	95% CI	p
Residential density									
Land use mix diversity									
Land use mix access									
Connectivity									
Walking infrastructure									
Cycling infrastructure									
Safety for cycling									
Aesthetics									
Safety for traffic				0.099(0.048)	0.005-0.193	0.040	0.116(0.051)	0.016-0.216	0.023
Safety for crime									
Convenience of recreation facilities									

Note: Due to multicollinearity between the variables “educational attainment mother” and “educational attainment father”, “self-efficacy internal” and “self-efficacy external” and “land use mix diversity” and land use mix access”, the variables “educational attainment father”, “self-efficacy internal” and “land use mix diversity” were excluded from further analyses. CI indicates confidence interval.

### Environmental correlates of cycling for transport during leisure time

Table 4 shows the results of the stepwise linear regression analyses with backward elimination concerning cycling for transport during leisure time. Residential density and cycling infrastructure were the only neighborhood built environmental attributes that were retained in the final model of the regression analysis adjusted for the demographic covariates (model 2). Residential density was found negatively but marginally associated (p = 0.076) with cycling for transport during leisure time, while cycling infrastructure was found positively but also marginally associated (p = 0.090) with cycling for transport during leisure time. In model 3 none of the neighborhood environmental attributes remained significant and all of the neighborhood environmental attributes were excluded from the final model.

**Table 4:** Results of the stepwise linear regression analyses with backward elimination concerning cycling for transport during leisure time

	**Cycling for transport during leisure time**								
	**MODEL 1**			**MODEL 2**			**MODEL 3**		
***Demographic characteristics***	ß (SE)	95% CI	p	ß (SE)	95% CI	p	ß (SE)	95% CI	p
Age (yrs)	0.066(0.026)	0.015-0.117	0.013	0.081(0.027)	0.028-0.134	0.003	0.068(0.027)	0.015-0.121	0.011
Gender (ref: male)	0.169(0.046)	0.079-0.259	<0.001	0.172(0.049)	0.076-0.268	<0.001	0.150(0.049)	0.054-0.246	0.002
Parental employment									
*Both unemployed (ref)*									
*One parent unemployed*	0.146(0.124)	−0.097-0.389	0.239	0.139(0.134)	−0.124-0.402	0.301	0.141(0.126)	−0.106-0.388	0.264
*Both employed*	0.177(0.122)	−0.062-0.416	0.148	0.131(0.132)	−0.128-0.390	0.320	0.164(0.124)	−0.079-0.407	0.187
Educational attainment mother									
*Less than high school (ref)*									
*Completed high school*									
*Completed college*									
*Completed University*									
Educational attainment father									
*Less than high school (ref)*									
*Completed high school*	−0.114(0.099)	−0.308-0.080	0.252	−0.065(0.107)	−0.275-0.145	0.542	−0.157(0.100)	−0.353-0.039	0.119
*Completed college*	−0.040(0.103)	−0.242-0.162	0.696	−0.010(0.111)	−0.228-0.208	0.929	−0.061(0.105)	−0.267-0.145	0.561
*Completed University*	−0.056(0.105)	−0.262-0.150	0.593	−0.004(0.112)	−0.224-0.216	0.964	−0.102(0.107)	−0.312-0.108	0.341
***Psychosocial factors***	ß (SE)	95% CI	p	ß (SE)	95% CI	P	ß (SE)	95% CI	P
Modeling							0.011(0.032)	−0.052-0.074	0.744
Social norm							0.009(0.024)	−0.038-0.056	0.698
Social support from family							−0.002(0.031)	−0.063-0.059	0.950
Social support from friends							0.030(0.029)	−0.027-0.087	0.309
Self-efficacy internal							0.061(0.038)	−0.013-0.135	0.142
Self-efficacy external									
Perceived benefits							−0.063(0.043)	−0.147-0.021	0.348
Perceived barriers							−0.044(0.047)	−0.136-0.048	0.106
***Perceived neighborhood environmental attributes***	ß (SE)	95% CI	p	ß (SE)	95% CI	P	ß (SE)	95% CI	p
Residential density				−0.001(0.001)	−0.003-0.001	0.076			
Land use mix diversity									
Land use mix access									
Connectivity									
Walking infrastructure									
Cycling infrastructure				0.065(0.038)	−0.009-0.139	0.090			
Safety for cycling									
Aesthetics									
Safety for traffic									
Safety for crime									
Convenience of recreation facilities									

Note: Due to multicollinearity between the variables “educational attainment mother” and “educational attainment father”, “self-efficacy internal” and “self-efficacy external” and “land use mix diversity” and land use mix access”, the variables “educational attainment mother”, “self-efficacy external” and “land use mix diversity” were excluded from further analyses. CI indicates confidence interval.

## Discussion and conclusion

Based on the results of the present study we might cautiously assume that the relationship between perceived neighborhood built environmental attributes and AT previously found in adults [34], is not totally comparable to the relationship found in Belgian adolescents. Among Belgian adolescents, the contribution of neighborhood built environmental perceptions to explain the variance in AT seems dependent of the purpose of AT. For AT during leisure time, the importance of the perception of the neighborhood environmental attributes seems rather negligible. After controlling for demographic characteristics and psychosocial factors, only the perception of a higher degree of traffic safety in the neighborhood was found to be associated with more walking for transport during leisure time. For cycling for transport during leisure time, none of the perceived neighborhood environmental attributes was found to be of importance. As to our knowledge this is one of the first studies to investigate the contribution of neighborhood built environmental attributes to active transport during leisure time among adolescents. Further research that focuses on AT during leisure time is therefore recommended. On the other hand, to explain AT to and from school the perception of the neighborhood built environmental attributes is important, even beyond the demographic characteristics and psychosocial factors. A shorter distance to school and perceiving neighborhoods to have connected streets, a lower degree of land use mix diversity, less infrastructure and a lower quality of the infrastructure for walking are found to be associated with more min/day AT to and from school. Furthermore, a higher degree of residential density and more safety for crime were also found to be marginally significantly associated with more AT to and from school. Within the literature focusing on active transport to school, the importance of the distance to school, the connectedness of neighborhood streets and the degree of residential density has been emphasized repeatedly [35-39]. These results highlight the need to build new schools in neighborhoods with a well-connected street network and a high degree of residential density. Furthermore, safety for crime was negatively associated with AT to and from school. Although this finding is somewhat counterintuitive, a lower perception of safety from crime is often associated with a higher residential density, which was positively associated with AT to and from school. Surprisingly, the availability and quality of walking infrastructure were negatively associated with AT to and from school. A possible explanation for this finding can be that adolescents prefer to take a route through parks, recreation domains or backstreets where no pavements or no well-maintained pavements are available instead of through busy city centers, which are usually characterized by paved and well-maintained sidewalks and bikelanes. Furthermore, we also found a negative association with land use mix diversity. A reason for this finding is not apparent. A more thorough examination of this issue is required.

Recently, two papers were published describing the association between neighborhood built environmental attributes and adolescents’ levels of PA in the same Belgian study sample. In the first paper [40], the same questionnaire was used to measure AT and the perception of the neighborhood built environmental attributes. In contrast to the results of the present study, cul-de-sacs and availability and quality of cycling infrastructure were found to be positively associated with AT. However, as this study did not make a distinction between the different types of AT, comparison needs caution. The second paper described the association of the objectively determined neighborhood walkability with walking and cycling for transport during leisure time and AT to and from school [24]. The results of this paper revealed no associations between objectively determined neighborhood walkability and self-reported AT. However, the difference in results can be attributed to the measurement method of the neighborhood environmental attributes. According to Ball et al. (2008), it is possible that objective assessments of built environmental attributes are indirectly associated with PA, whereas perceptions of environmental attributes have a more direct influence on PA.

A possible explanation for the distinction in the importance of neighborhood built environmental attributes to explain AT to and from school and AT for other purposes, might be that parents of young adolescents still play an important role in determining adolescents’ exposure to factors that are favorable or unfavorable to PA [41]. Young adolescents are often dependent on parental rules governing travel and destination choices. During the week most of the adolescents’ parents are both employed which makes it very difficult to drive their children from home to school and pick their children up after school. Belgian adolescents between 13 and 15 years are not allowed to drive cars nor mopeds and often have few alternative transport modes besides walking and cycling to travel to and from school. The distance to school and the perception of the adolescents of other built environmental attributes can then be of importance to explain AT to and from school in adolescents. In contrast, after school hours and during the weekends, the parents more often have the time and opportunity to transport their children. Therefore, it is possible that adolescents’ AT during their leisure time is more dependent on parental perceptions of neighborhood environmental attributes. Furthermore, during leisure time, the adolescents more often have the choice to be driven by their parents to their destinations or to use AT. It is possible that other factors (e.g. the possibility to be independent) rather than their perception of the environmental attributes are of greater importance in this choice.

Second, the items in the questionnaire concerning the environmental perceptions were related to the adolescents’ own neighborhood. Because a large part of adolescents are involved in sports and other leisure activities, adolescents’ AT during leisure time involve also the route from home to sport or leisure facilities. Consequently, built environmental characteristics of these environments may need to be taken into account. Thus, it would be advisable for future research to also include built environmental characteristics of other routes and destinations where adolescents often travel to during leisure time, such as their friend’s house, sport facilities or parks. Finally, the absence of associations can be attributable to the relatively high activity friendliness of Belgian neighborhoods. In contrast to other continents and countries such as the USA or Australia the built environment in Belgium is quite supportive for walking and cycling [42]. Consequently, this will be reflected in the variability in AT.

When considering the present study results, it should be taken into account that due to the cross-sectional design no inferences on causality can be made. Secondly, as we relied on self-report, our data may suffer from reporting bias. Third, certain neighborhood environmental factors (e.g. quality and attractiveness of parks and neighborhood sport facilities [43,44]) that were not included in our questionnaire may also be of importance in explaining AT among adolescents. These factors should be investigated more thoroughly in the future. Fourth, comparison of gender distribution, parental employment status and educational level with data from the Belgian National Institute of Statistics showed that the study sample was comparable for gender, but the parents were more likely to be highly educated and employed. This may limit the generalizability of our findings. Strengths of the present study are the large study sample and the use of validated questionnaires to measure built environmental perceptions and AT.

For public health researchers, organizers and providers and policy makers involved in the development of interventions to promote PA, the results of the present study provide evidence that among Belgian adolescents, the perceptions of neighborhood built environmental attributes might be of importance for AT to and from school. Consequently, changing the perception of built environment attributes by awareness-raising initiatives may be effective in the promotion of AT tot and from school but not in the promotion of AT during leisure time. Of course, when considering these results and conclusions, it should be taken into account that they refer to the overall Belgian adolescent population and that they are possibly not applicable for specific subgroups. For example for groups that are most difficult to reach (i.e. adolescents living in socio-economically disadvantaged neighborhoods with less positive scores on psychosocial factors) positive environmental perceptions might help in overcoming personal barriers towards PA. Further research in specific subgroups is therefore needed.

## Competing interests

The authors declare that they have no competing interests.

## Authors’ contributions

FDM coordinated the data collection, assisted in the recruitment of the participants, conducted the statistical analyses and drafted the manuscript. GC, DVD, IDB and BD participated in the interpretation of the data, revised the draft versions of the manuscript and provided critical comments during the process. All authors read and approved the final manuscript.
